# Naming under constraint: gender, care and symbolic power in family life

**DOI:** 10.3389/fsoc.2026.1850315

**Published:** 2026-06-17

**Authors:** Patrícia Albergaria-Almeida

**Affiliations:** Departamento de Educação e Ensino à Distância, Universidade Aberta, Lisbon, Portugal

**Keywords:** care and mental load, consent, delayed articulation, gender inequality, naming practices, symbolic power

## Abstract

This article proposes a reconceptualisation of children’s surname attribution as a gendered site of symbolic power within heterosexual family life. While naming is commonly framed as a private and consensual decision, feminist scholarship has shown that family practices are structured by persistent asymmetries of care, authority, and recognition. Yet the symbolic dimension of naming remains underexamined. Building on feminist theories of care, symbolic violence, and situated knowledge, this article introduces the concept of naming under constraint to analyse how naming decisions may be produced under conditions that limit deliberation and agency. Drawing on a case in Portugal, it shows how a naming decision taken immediately after childbirth—under bodily vulnerability, institutional urgency, and unequal care relations—becomes stabilised as a consensual outcome. The article further develops the notion of delayed articulation to theorise the temporal gap between living inequality and naming it as such, highlighting how care-intensive life stages constrain feminist critique. By shifting attention from naming as a neutral outcome to naming as a situated process, this article contributes to sociological debates on gender, care, and symbolic power, and highlights the importance of attending to the temporal and relational conditions under which inequality becomes intelligible.

## 1 Introduction

The naming of a child is commonly treated as a private, intimate, or administrative act. Within families, it is often framed as a matter of tradition, personal preference, or legal compliance rather than as a site of social power. Yet names do more than identify individuals. They organise lineage, stabilise belonging, and symbolically position subjects within gendered and generational orders. Surnames, in particular, make kinship publicly legible: they signal continuity, inheritance, affiliation, and recognition. For this reason, the attribution and ordering of children’s surnames offer a revealing site for examining how gendered power may be reproduced through everyday family practices that often appear minor, consensual, or self-evident.

Sociological and feminist scholarship has long shown that heterosexual family life remains structured by persistent inequalities in care, responsibility, authority, and recognition (Coltrane, 1996; Risman, 2004). Classic work on the “second shift” and emotion work has demonstrated how family life depends on forms of labour that are unequally distributed and frequently naturalised as women’s responsibility (Hochschild, 1979; Hochschild and Machung, 2003). More recent work on cognitive labour and mental load has further shown that inequality is not limited to visible tasks, but also includes anticipation, planning, remembering, and managing everyday life (Daminger, 2019). These asymmetries are not merely practical. They also shape the symbolic organisation of family life, including whose lineage is prioritised, whose contribution is recognised, and whose position becomes publicly inscribed as central.

Existing research on naming practices has made important contributions to this discussion. Studies of marital name change have shown that surnames function as a window into gender attitudes, marital identity, and the persistence of gendered expectations within couple relationships (Hamilton et al., 2011). Research on children’s surnames has demonstrated that decisions about what to call children are often shaped by moral dilemmas, assumptions about family continuity, and the continued symbolic force of paternal lineage (Johnson and Scheuble, 2002; Nugent, 2010). More recent work on couple identity and naming has further highlighted how naming decisions may appear collaborative while remaining structured by gendered inequalities and power (Sue et al., 2024). Taken together, this literature makes clear that naming is not a neutral domain. It is a socially organised practice through which gender, kinship, and family authority are made visible and durable.

This article builds on that scholarship, but shifts the analytical focus from naming outcomes to naming processes. Rather than asking only which surname is chosen, or what that choice reveals about gender attitudes, it asks under what relational, bodily, institutional, and temporal conditions naming decisions are made. This shift matters because decisions that appear formally consensual may be produced within circumstances that constrain deliberation, resistance, or refusal. In such cases, the language of choice may obscure the social conditions under which agreement becomes possible.

The article develops this argument through a situated case in Portugal, a context in which the attribution and ordering of children’s surnames is legally flexible but culturally structured. Portuguese law allows parents considerable discretion in the selection and order of surnames. However, it remains customary for the father’s surname to occupy the final position, widely recognised as the symbolically dominant one. This legal permissiveness is sociologically significant. Because paternal precedence is not formally imposed by law, its persistence cannot be explained simply as legal constraint. Instead, it invites closer attention to the cultural, relational, and institutional mechanisms through which patrilineal naming remains normalised as tradition, preference, or common sense.

The analysis draws on a single situated vignette from heterosexual family life: a naming decision taken in the immediate aftermath of childbirth, while the mother was still physically vulnerable and undergoing medical intervention. The father proposed a full name for the newborn, placing his surname in the final position; the mother assented. At the level of interaction, the exchange appeared consensual. No explicit conflict occurred, no objection was voiced, and no coercion was visible. Yet the conditions under which this assent was produced are analytically decisive. The decision was made under bodily vulnerability, institutional urgency, and pre-existing asymmetries of care and authority. These conditions complicate any straightforward reading of agreement as free, equal, or fully deliberative consent.

To analyse this dynamic, the article introduces the concept of *naming under constraint*. The concept does not refer to overt coercion or legal imposition. Rather, it designates the production of naming decisions under conditions that limit the practical possibility of questioning, negotiating, or refusing. Naming under constraint foregrounds how symbolic power may operate through timing, normalisation, and relational asymmetry, rather than through explicit domination. In this respect, the concept resonates with analyses of hidden power in marriage, where existing arrangements may be maintained not through open conflict, but through non-decision, silence, deflection, and the unequal distribution of the capacity to make claims (Komter, 1989). It also builds on theories of symbolic violence, which show how socially produced hierarchies become misrecognised as natural, legitimate, or not worth contesting (Bourdieu, 2001).

The article also develops the concept of *delayed articulation* to theorise the temporal gap between living inequality and naming it as such. The naming episode analysed here did not immediately appear as an object of feminist critique. Its significance became intelligible retrospectively, after temporal distance, accumulated experience, and conceptual resources made it possible to recognise the decision as part of a broader pattern of gendered power. This delay is not treated as a failure of awareness. Rather, it is understood as a structurally produced epistemic condition. Care-intensive life stages, including pregnancy, childbirth, and early motherhood, constrain not only time and energy, but also the cognitive and symbolic space required to formulate critique. In this sense, delayed articulation extends feminist insights into the temporal and situated character of consciousness, critique, and the transformation of personal experience into political knowledge (Hanisch, 2006; Ahmed, 2017).

The two concepts—*naming under constraint* and *delayed articulation*—are analytically connected but address different moments of the same process. Naming under constraint refers to the conditions under which the naming decision was produced; delayed articulation refers to the later temporal and epistemic process through which that decision became intelligible as a gendered inequality. The first concept therefore concerns the situated production of assent, while the second concerns the deferred recognition and naming of the conditions under which that assent was given.

As a Perspective article, the aim is not to claim empirical representativeness from a single case. The vignette is used as a theoretically productive entry point for conceptual analysis. Its value lies in making visible mechanisms that are often difficult to apprehend precisely because they are ordinary, intimate, and culturally normalised. By bringing naming into sociological view as a situated process, the article contributes to debates on gender, family power, care, consent, and symbolic inequality. It argues that naming practices are not merely residual traditions or private preferences, but consequential sites where gendered hierarchies are enacted, stabilised, and transmitted across generations.

## 2 A situated vignette: naming under constraint

### 2.1 Methodological note: auto-socio-biographical analysis

The analysis developed in this article is grounded in an auto-socio-biographical approach, understood here as a mode of feminist sociological inquiry in which situated personal experience is examined as analytically productive material rather than as private anecdote. The empirical material consists of longitudinal reflective notes written at different moments across and after pregnancy, childbirth, and early motherhood. Some notes were produced close to the events described, while others were written later, when temporal distance and conceptual resources made it possible to revisit the experience as part of a broader pattern of gendered care, symbolic recognition, and family power.

The reflective notes were not treated as representative empirical data, nor are they mobilised to establish the prevalence of a pattern across families. Instead, they are used as situated material for conceptual analysis. Their selection was guided by their relevance to the mechanisms examined in this article: surname attribution, care asymmetry, bodily vulnerability, institutional timing, consent, constraint, and the delayed recognition of inequality. The analytical process involved rereading the notes in dialogue with feminist sociology, theories of care and mental load, symbolic violence, and work on naming practices, in order to identify how an apparently ordinary family decision could be understood as a site of symbolic power.

This methodological choice is consistent with feminist, auto-socio-biographical, and auto-ethnographic traditions that regard situated experience as a legitimate entry point into the analysis of social structures, provided that it is reflexively examined and connected to broader sociological processes (Bourdieu, 2004; Stanley, 1992; Harding, 1991). As Bourdieu (2004) argues in *Sketch for a Self-Analysis*, reflexive engagement with one’s own social position can constitute a rigorous sociological method when systematically connected to structural analysis. Feminist standpoint epistemology similarly contends that situated knowledge, far from being a limitation, may provide a distinctive entry point into mechanisms of power that are often invisible from more detached vantage points (Harding, 1991; Hill Collins, 1990). The aim is therefore not to move from one case to empirical generalisation, but to use one situated case to develop concepts that may illuminate mechanisms of gendered power that are difficult to identify precisely because they operate through ordinary, intimate, and culturally normalised practices.

The vignette analysed below is therefore presented as a theoretically generative case through which to examine how naming decisions may be produced, stabilised, and retrospectively understood within gendered conditions of care, vulnerability, and symbolic asymmetry.

### 2.2 Legal and cultural context of the naming vignette

In Portugal, the attribution and ordering of children’s surnames is legally flexible but culturally structured. While parents are free to choose the combination and order of surnames, it is customary for the father’s surname to occupy the final position, widely recognised as the symbolically dominant one. This convention is typically reproduced without explicit negotiation and often appears as a neutral or self-evident aspect of family life. This apparent neutrality is sociologically significant, since research on children’s surnames has shown that naming decisions are often shaped by gendered expectations, moral dilemmas, assumptions about family continuity, and the symbolic force of paternal lineage (Johnson and Scheuble, 2002; Nugent, 2010; Pilcher, 2017).

Legally, therefore, Portuguese naming rules do not impose a fixed order of maternal and paternal surnames. Under Article 1875 of the Portuguese Civil Code, a child may use surnames from both parents or from only one of them, and the choice of the child’s forenames and surnames belongs to the parents; in the absence of agreement, the decision is referred to the court in accordance with the child’s interests (Portuguese Civil Code, 1966, Article 1875). This legal flexibility is analytically important because it means that paternal final-position naming cannot be explained simply as a formal legal requirement. Rather, its persistence points to the cultural and symbolic force of convention. In this context, the final surname position often functions as the most publicly salient marker of family lineage and intergenerational continuity, which helps explain why the ordering of surnames may carry symbolic weight even when it appears administratively minor.

The case involves a heterosexual couple navigating naming decisions across two successive births. At the birth of the first child, the dominant convention was followed without reflection: the mother’s surname preceded the father’s, which occupied the final position. At that moment, the decision did not register as problematic. It was absorbed into the broader experience of early motherhood, marked by physical recovery, emotional reorganisation, and the immediate demands of care. The absence of questioning should not be read as strong agreement or indifference. Rather, it points to the conditions under which family practices become naturalised, especially during care-intensive life stages when critical distance is difficult to sustain.

The emergence of questioning occurred during the second pregnancy. By this point, the uneven distribution of care had become more visible. Responsibility for anticipating needs, organising childcare, and managing everyday life had fallen disproportionately on the mother, reflecting patterns widely documented in the literature on gendered care, cognitive labour, and mental load (Hochschild and Machung, 2003; Daminger, 2019). Within this context, the order of surnames began to surface as a source of unease:

“I keep returning to the question of the surname. I don’t know why it feels so important, but it does. I do everything: the baby, the house, the planning, the nights. And yet it seems obvious that it should be his name that comes last. I find myself asking why.” (Reflective note, second pregnancy)

This moment captures a form of inequality that is fully lived but not yet fully articulated. The discomfort does not initially take the form of a political claim but emerges as a persistent disturbance. The juxtaposition between intensive care labour and the symbolic prioritisation of paternal lineage reveals a misalignment between responsibility and recognition that has not yet found a stable language. The issue is not only that the paternal surname is conventionally prioritised, but that this prioritisation becomes harder to sustain as neutral when everyday care is unequally carried.

Attempts to question the naming convention did not result in explicit conflict. Instead, they were met with hesitation, deflection, and eventual non-resolution. The possibility of changing the order of surnames was raised but not pursued. The issue remained suspended, illustrating how existing arrangements can be maintained through inertia rather than overt enforcement. This dynamic resonates with analyses of hidden power in marriage, where power operates not only through explicit decision-making, but also through non-decision, avoidance, silence, and the unequal capacity to define what counts as a legitimate issue for discussion (Komter, 1989).

The naming decision for the second child ultimately took place in the immediate aftermath of childbirth. Approximately 10 min after delivery, while the mother was still undergoing medical suturing following an episiotomy, the question of the child’s name was raised. Hospital procedures required that the newborn be identified, and the decision could no longer be deferred.

In this context, the father proposed a full name for the child, placing his surname in the final position. The proposal was framed as confirmation rather than as an open question. The mother assented.

“I was still in the delivery room, only a few minutes after the birth. He was in the adjacent room with the doctor and the nurse and came to ask me whether the baby’s name was A B C, with C being his surname. At that moment, exhausted and being stitched after the delivery, I said yes, just as I would have said yes to any name that had been proposed.” (Reflective note, childbirth)

At the level of interaction, this exchange appeared consensual. No conflict was expressed, and no coercion was visible. Yet the conditions under which the decision was made are analytically decisive. The mother’s capacity to deliberate or resist was materially constrained by pain, exhaustion, and ongoing medical intervention. Institutional urgency compressed the temporal window for decision-making, while prior asymmetries in care and authority shaped the terms under which the proposal was made.

The assent given in this context cannot be understood as a simple expression of agreement. Rather, it reflects a form of assent produced under conditions that limited deliberation, negotiation, and contestation. What is subsequently stabilised as a neutral and consensual outcome is, in fact, shaped by the convergence of bodily vulnerability, institutional timing, and relational asymmetry. The sociological significance of the episode lies precisely in the way no overt domination is required for paternal symbolic precedence to be reproduced. The decision appears ordinary because it aligns with convention; it appears consensual because no resistance is voiced; and it becomes durable because the conditions that constrained resistance are subsequently effaced.

This vignette is therefore not presented as evidence of a general pattern, but as an analytically revealing instance of how naming decisions may be produced within structured conditions that align symbolic authority with paternal lineage, even in contexts formally characterised by choice and equality. It foregrounds a process through which a naming outcome may come to appear natural, consensual, and inconsequential, while obscuring the bodily, institutional, and relational constraints under which it was produced.

## 3 Naming under constraint

The vignette presented above invites a shift in how naming practices are conceptualised. Rather than treating naming as a discrete decision or as the outcome of individual preference, it suggests the need to analyse naming as a situated process shaped by the conditions under which consent is produced.

To capture this dynamic, I propose the concept of naming under constraint. This concept does not refer to overt coercion or legal imposition. Instead, it designates a form of constraint that operates through the alignment of relational asymmetries, embodied vulnerability, institutional timing, and culturally sedimented expectations, structuring what can be reasonably questioned, negotiated, or refused in a given moment.

Feminist and sociological analyses have long shown that power is not only exercised through explicit domination, but also through processes of normalisation, misrecognition, and non-decision. Symbolic violence, in Bourdieu’s sense, functions by rendering historically and socially produced arrangements as natural, self-evident, and beyond contestation (Bourdieu, 2001). Naming practices exemplify this dynamic. The prioritisation of paternal lineage often appears as tradition, preference, or family continuity, rather than as an effect of gendered power relations.

At the same time, naming under constraint resonates with analyses of hidden power in marriage. Komter (1989) shows that power within intimate relationships may operate not only through overt conflict or explicit decision-making, but also through avoidance, silence, non-decision, and the unequal capacity to define what counts as a legitimate issue for negotiation. In the vignette analysed here, the possibility of reversing the surname order was not openly refused, but deferred and left unresolved until the decision reappeared in a moment when contestation was least feasible. The maintenance of paternal symbolic precedence therefore did not require explicit enforcement. It was sustained through hesitation, inertia, and the unequal capacity to keep the issue from becoming fully negotiable.

This is particularly important in relation to naming because existing scholarship has shown that surnames are not merely labels, but symbolic resources through which gender, kinship, couple identity, and family continuity are negotiated. Research on children’s surnames has highlighted the moral dilemmas surrounding naming when parents’ surnames differ, as well as the continued predominance of paternal surnames as markers of family legitimacy and continuity (Johnson and Scheuble, 2002; Nugent, 2010). More recent work has further shown that naming decisions may appear collaborative while remaining structured by gendered asymmetries (Sue et al., 2024). Naming under constraint extends this insight in a specific direction. Where Sue et al. (2024) focus on the interactional and discursive work through which couples co-construct a shared naming identity—showing how collaboration can coexist with gendered asymmetry—the concept proposed here shifts attention to the conditions under which that collaborative framing becomes available in the first place. It foregrounds the bodily, temporal, and institutional circumstances that precede and shape the naming moment, suggesting that what appears as joint decision-making may already be structurally foreclosed before any explicit negotiation begins. The contribution is thus not to dispute the relational dynamics identified by Sue et al. (2024), but to ask what lies upstream of them: the prior asymmetries of care, vulnerability, and symbolic authority that determine who is in a position to deliberate at all.

The notion of naming under constraint therefore foregrounds the temporal and embodied conditions under which naming decisions are made. In the case discussed, the decision took place in a moment characterised by postpartum vulnerability, physical exhaustion, and institutional urgency. These conditions are not incidental. They actively shaped the possibility of deliberation. The mother’s assent was not extracted through force, but produced in a context where resistance would have required disproportionate effort, disrupted an institutional script, and challenged a culturally normalised expectation.

At the same time, these immediate conditions were embedded within longer-term relational asymmetries. The unequal distribution of care, cognitive labour, and responsibility preceding the moment of naming established a pattern in which one actor was more deeply invested in sustaining everyday life, while the other retained greater symbolic authority at a key decision point. This misalignment was not resolved at the moment of naming; rather, it was condensed within it. The name became a symbolic site where unequal care and unequal recognition met.

Naming under constraint therefore offers an analytical lens for examining how symbolic decisions may be produced at the intersection of multiple layers of inequality: relational asymmetry, reflected in unequal distributions of care and authority; bodily vulnerability, particularly in contexts such as childbirth; institutional timing, which compresses decision-making into narrow temporal windows; and cultural conventions that make paternal precedence appear natural before any explicit discussion begins. These dimensions do not operate independently. Their convergence creates conditions in which contestation becomes difficult, costly, or practically unfeasible.

Importantly, the outcome of such processes is not necessarily experienced as imposed. Once stabilised, naming decisions can be retrospectively narrated as consensual and unproblematic. The conditions of constraint are effaced, replaced by a narrative of agreement that aligns with dominant understandings of family decision-making as based on choice, collaboration, and mutual consent. This retrospective smoothing is central to the durability of symbolic power. It allows an unequal process to become legible as an ordinary family decision.

This is precisely how symbolic power operates most effectively: not by eliminating agency, but by shaping the conditions under which agency is exercised. Naming under constraint thus allows us to reconceptualise consent not as a binary—present or absent—but as a situated and relationally structured process. The question is not simply whether agreement was given, but under what bodily, relational, institutional, and symbolic conditions agreement became the available response.

It is important to distinguish naming under constraint from coercion, absence of agency, or the mere absence of resistance. Coercion implies an explicit threat, force, or direct imposition; that is not the dynamic at issue here. Nor does naming under constraint mean that the subject has no agency or that agreement is simply false. Rather, it refers to a form of assent given in circumstances where the practical possibility of deliberation, objection, or negotiation is substantially reduced. In this sense, the mother’s “yes” is neither treated as meaningless nor read as fully equivalent to a decision made under conditions of temporal distance, bodily ease, and relational symmetry. The point is not to invalidate consent, but to show that consent is situated: it may be relationally, temporally, institutionally, and bodily conditioned. Naming under constraint therefore occupies an analytical space between free, symmetrical agreement and overt coercion, drawing attention to the social conditions under which assent becomes the most available response.

By shifting attention from naming as an outcome to naming as a process, this concept contributes to broader debates on gender, family power, and symbolic inequality. It suggests that symbolic practices—often treated as minor or secondary—may constitute key sites where gendered hierarchies are enacted, stabilised, and rendered durable. Naming under constraint helps make visible the quiet mechanisms through which formally available choices may reproduce unequal arrangements, especially when those choices are made within contexts structured by care, vulnerability, and hidden power.

## 4 Delayed articulation and feminist knowledge

The analysis developed so far raises a further question: why do situations such as the one described often remain unchallenged at the moment in which they occur, only becoming intelligible as problematic retrospectively?

This question marks the point at which the second concept developed in this article becomes necessary. If naming under constraint refers to the relational, bodily, institutional, and symbolic conditions under which assent becomes the available response, delayed articulation refers to the temporal and epistemic process through which those conditions later become recognisable as inequality. The two concepts are therefore analytically connected. Naming under constraint concerns the situated production of the decision; delayed articulation concerns the deferred recognition of what was at stake in that decision.

Delayed articulation thus refers to the temporal gap between the lived experience of inequality and its recognition and naming as such. Rather than treating this delay as a failure of awareness, reflexivity, or political consciousness, the concept foregrounds the social and material conditions that structure when and how critique becomes possible.

The naming episode analysed here did not become immediately intelligible as a site of gendered symbolic power. At the time, discomfort was present, but it did not yet take the form of a stable claim. It appeared instead as unease, irritation, or disproportionate concern with something that seemed socially minor. This matters because symbolic power often operates precisely by making inequality difficult to recognise as inequality. What later becomes visible as constraint may initially be lived as confusion, hesitation, or self-doubt.

Feminist scholarship has long emphasised that gendered inequality is not always immediately recognised as structural. The insight captured in the slogan “the personal is political” points to the processes through which experiences initially lived as private, excessive, or idiosyncratic become intelligible as part of broader social arrangements (Hanisch, 2006). Ahmed’s work on feminist consciousness is also relevant here, insofar as it shows how becoming attuned to inequality often involves a retrospective re-reading of ordinary experiences that had previously been absorbed into the background of everyday life (Ahmed, 2017).

Delayed articulation extends these insights by foregrounding the temporal and care-related conditions of critique. Care-intensive life stages such as pregnancy, childbirth, and early motherhood are characterised by heightened demands on time, attention, and emotional resources. These conditions limit not only the capacity for reflection, but also the possibility of sustaining critical distance from the arrangements that structure everyday life. Experiences that may later appear as instances of inequality are, in the moment, often absorbed into the practical and affective work of maintaining care, continuity, and family stability.

In this sense, delayed articulation is not simply a temporal delay; it is a structurally produced epistemic condition. The capacity to name inequality is unevenly distributed, shaped by the organisation of care, dependency, cognitive labour, and emotional responsibility within family life. Work on the second shift, emotion work, and cognitive labour has shown that women’s responsibilities often extend beyond visible tasks to include anticipation, affective management, and the maintenance of relational stability (Hochschild, 1979; Hochschild and Machung, 2003; Daminger, 2019). These responsibilities affect not only what women do, but also when and how they are able to interpret what is happening to them. The demands of emotion work do not only redistribute labour; they also redistribute epistemic availability. To be responsible for anticipating, managing, and absorbing the emotional needs of others is to have less cognitive and temporal space available for the kind of distancing that structural critique requires. In this sense, Hochschild’s (1979) analysis of feeling rules and emotional labour provides an important but underexplored resource for feminist epistemology: the unequal distribution of emotional labour is also an unequal distribution of the conditions under which inequality can be recognised and named.

This temporal dimension is particularly relevant for understanding symbolic violence. Because symbolic power operates through normalisation and misrecognition, it does not typically present itself as domination (Bourdieu, 2001). Instead, it is lived as common sense, as what “makes sense” within a given relational and cultural context. The absence of immediate contestation should therefore not be interpreted as evidence of genuine agreement. It may instead reflect the conditions under which recognition becomes possible or remains deferred.

Delayed articulation also challenges choice-based accounts of family decision-making. Such accounts often assume that individuals are able to recognise, evaluate, and contest unequal arrangements in real time. Yet the case analysed here suggests that this assumption is inadequate. If inequality becomes intelligible only retrospectively, then consent cannot be fully understood by examining the moment of agreement alone. The temporal conditions of recognition must also be taken into account. What is agreed to in one moment may only later become recognisable as having been agreed to under constraint.

Importantly, delayed articulation is not only a limitation; it is also a condition of feminist knowledge production. The retrospective interpretation of experience allows for the identification of patterns, the development of concepts, and the articulation of critique. In this sense, feminist knowledge often emerges not in the immediacy of experience, but through processes of temporal distancing that make structural analysis possible. The delay is therefore not merely a lag between experience and understanding. It is part of the process through which experience becomes sociologically and politically intelligible.

By foregrounding delayed articulation, this article contributes to debates on the relationship between experience, temporality, and knowledge in feminist sociology. It suggests that the capacity to transform lived experience into sociological insight is itself conditioned by the organisation of everyday life, and particularly by the unequal distribution of care. In doing so, the concept helps explain why symbolic forms of gendered power may remain unchallenged when they occur, while later becoming central to feminist critique.

## 5 Discussion: implications and future directions

The concepts developed in this article—naming under constraint and delayed articulation—invite a reconsideration of how gendered inequalities are produced, stabilised, and later recognised within family life. Taken together, they link the situated dynamics of decision-making to the temporal conditions of feminist recognition: naming under constraint foregrounds how assent may be produced under unequal relational, bodily, institutional, and symbolic circumstances, while delayed articulation foregrounds how the significance of that assent may only become intelligible retrospectively. Existing research has shown that surname practices are deeply implicated in gender attitudes, couple identity, moral dilemmas around children’s names, and the persistence of paternal lineage (Hamilton et al., 2011; Johnson and Scheuble, 2002; Nugent, 2010; Sue et al., 2024). The contribution of this article is to extend that discussion by shifting attention from naming outcomes to the relational, embodied, institutional, and temporal processes through which naming decisions may be produced.

Naming decisions may, of course, be understood by participants as tradition, pragmatic convention, mutual preference, or family continuity. The point is not to deny these meanings, nor to suggest that they are necessarily insincere. Rather, the question is how such meanings become available as common-sense explanations within gendered relational contexts, and how they may obscure the conditions under which some options become easier to imagine, propose, or accept than others. Engaging these alternative interpretations is therefore central to the argument: it is precisely because naming can be experienced as ordinary, practical, or mutually agreed that it requires sociological attention as a possible site of symbolic power.

A first implication concerns the limits of choice-based frameworks. Much contemporary analysis of family decision-making assumes that the availability of alternatives is sufficient to ensure equality. From this perspective, if parents are legally free to choose the order of surnames, then the persistence of patrilineal naming may be interpreted as a matter of preference rather than power. However, the analysis presented here suggests that such an interpretation overlooks the conditions under which choices are made. When decisions are taken within contexts marked by asymmetries of care, bodily vulnerability, institutional urgency, and culturally normalised expectations, the notion of free and equivalent choice becomes analytically insufficient. Naming under constraint helps make visible how apparently neutral decisions may reproduce structured inequalities precisely because they are taken under unequal conditions.

A second implication concerns the relationship between care and symbolic authority. Sociological research has extensively documented the unequal distribution of care work, emotion work, cognitive labour, and mental load within heterosexual families (Hochschild, 1979; Hochschild and Machung, 2003; Daminger, 2019). Yet less attention has been paid to how these inequalities shape symbolic domains, such as naming. The case examined here suggests that responsibility for care and responsibility for symbolic inscription do not necessarily align. On the contrary, those who bear the greater burden of care may have less capacity to influence decisions that carry long-term symbolic weight. This disjuncture highlights the need to analyse symbolic practices not as secondary or merely expressive domains, but as central sites where gendered hierarchies are consolidated and made durable.

A third implication relates to power within intimate relationships. The analysis suggests that the reproduction of paternal symbolic precedence does not require overt domination or explicit refusal. It may instead operate through hesitation, deflection, non-resolution, and the unequal capacity to define what counts as a legitimate issue for discussion. In this respect, naming under constraint resonates with analyses of hidden power in marriage, where existing arrangements are maintained not only through decisions, but also through non-decisions and the prevention of certain matters from becoming negotiable (Komter, 1989). This is particularly important for understanding naming practices, since decisions that appear collaborative or consensual may still be structured by unequal capacities to deliberate, insist, defer, or resist.

A fourth implication concerns temporality and the production of critique. The concept of delayed articulation challenges the assumption that inequality, if present, will be immediately recognised and contested. Instead, it suggests that recognition is itself a temporally structured process, shaped by the organisation of everyday life and the unequal distribution of cognitive and emotional resources. This has important consequences for how agency and consent are conceptualised. The absence of immediate resistance cannot be straightforwardly interpreted as agreement; it may instead reflect the conditions under which critique is rendered difficult, costly, or deferred. What appears consensual in the moment may only later become intelligible as having been produced under constraint.

Taken together, these implications suggest that the reproduction of gender inequality within families cannot be fully understood through frameworks that prioritise formal rights, individual preferences, or explicit forms of domination. Rather, it requires attention to the interplay between relational asymmetries, embodied conditions, institutional arrangements, cultural conventions, and temporal processes.

The analysis is necessarily limited by its reliance on a single retrospective and self-reflexive case. It does not claim empirical prevalence, representativeness, or direct transferability across family contexts. Its contribution lies instead in developing an analytical model that may be tested, refined, extended, or challenged in future comparative and empirical research. This limitation is also part of the article’s methodological positioning: the situated character of the case is not treated as a basis for generalisation, but as a means of making visible mechanisms that may otherwise remain obscured within ordinary family practices.

Future research could build on this analysis in several directions. Comparative studies across different legal and cultural contexts would help to examine how naming practices are shaped by varying configurations of constraint and possibility. Further work could also explore how alternative naming arrangements are negotiated and sustained, and under what conditions they become socially intelligible or legitimate. More broadly, the concepts proposed here may be extended to other domains of family life—such as decisions around schooling, residence, caregiving arrangements, or the allocation of family responsibilities—where consent appears unproblematic but is structured by unequal conditions.

By bringing naming into sociological view as a situated and consequential practice, this article contributes to a broader reorientation in the study of family life. It suggests that everyday decisions—particularly those that appear minor, private, or self-evident—constitute key sites where gendered power is enacted, stabilised, and transmitted across generations. Attending to these moments does not simply add detail to existing accounts of inequality; it reshapes how inequality itself is understood.

## Data Availability

The raw data supporting the conclusions of this article will be made available by the authors, without undue reservation.
